# Using Knowledge Management to Improve the Effectiveness of Data Fusion Centers

**DOI:** 10.4018/ijkm.297609

**Published:** 2022-01-01

**Authors:** Logan Willman, Murray Eugene Jennex, Eric G. Frost

**Affiliations:** San Diego State University, USA; West Texas A&M University, USA; San Diego State University, USA

**Keywords:** Data Fusion, Knowledge Management, Knowledge Sharing, Knowledge Visualization, Structured Analytic Technique

## Abstract

Data fusion centers have been around since the 9/11 terrorist attacks but have been shackled with an underperforming label. Products from fusion centers often do not support the mission of the organizations supporting the centers. This paper addresses the questions of the historical barriers to information sharing and how fusion centers can become more efficient and effective in this role. The research and experience of the authors are viewed through a knowledge management lens to propose using structured analytic technique exercises coupled with applying knowledge management tools and concepts to improve knowledge flow and the relevance and quality of the intelligence products produced.

## 1. INTRODUCTION

State and local fusion centers were initially created in the wake of the 9/11 Commission Report to help “connect the dots” of seemingly disparate threat indicators and, through information sharing, aid the federal counterterrorism mission at the grassroots level. Today, this system consists of 79 centers: Forty-two states and territories each has single primary centers, while a dozen states (notably Texas and California) have additional “recognized” centers to cover key regions (Department of Homeland Security, 2019). While fusion centers have assisted in identifying leads for what would become counterterrorism investigations, their day-to-day utility is increasingly an all-crimes, all-hazards mission (Rollins, 2008). Fusion centers are made up of personnel from multiple law enforcement organizations at the state and local levels and federal agencies, and may include civilian analysts as well.

The value of fusion centers is far more than the sum of their parts; and recognizing the many non-counterterrorism functions they fulfill is important in demonstrating this. This is not always easy to validate, and fusion centers have come under significant criticism and scrutiny since their inception. The criticism that fusion centers receive stems from the poorly defined and loosely understood method in which they are evaluated. Assessments of fusion centers are based on metrics having to do with percentage of products answering national requirements, customers’ satisfaction with fusion center products, numbers of products produced, and the perception of supported agencies (DHS, 2018). The difficulty with having a report card tied to arbitrary production metrics rather than results is that the focus inevitably shifts to meet expected numbers instead of meeting expected results. An example consequence of national grading requirements is that only 10% of the fusion center distributable products created in 2016 addressed state/local customer information needs (DHS, 2016).

The goal of any endeavor in intelligence is to increase the understanding of a particular problem set within a target audience. Whether the target audience consists of decision makers, action takers, or those who are only tangentially affected, the reason for conducting the function of intelligence is to analyze information in order to answer the question, “so what?” (Fingar, 2011). Quite often that function is much easier said than done due to both the subjectivity and complexity involved with interpreting partial and incomplete data to draw meaningful conclusions. As with many complex processes, the function of intelligence is at the same time highly structured and very fluid. Governed by recognized standards at the national level, organizations that belong to and adhere to these Intelligence Community Directives (ICDs) establish strict procedures to review and handle quality control for any analytical products they create (Office of the Director of National Intelligence, 2020). Likewise, these standards guide every part of the analytical process.

The member organizations of the Intelligence Community (IC) each operate under specific charters, whose actions and foci are made distinct by varying authorities within the mission of the organization. Because many aspects of those specific missions have a domestic aspect, even more complexity is introduced as protections are enacted to preserve the privacy and respect the First Amendment Rights of U.S. Persons (Rollins, 2008). Additionally, the domestic realm is where the national security responsibilities of federal agencies most often intersect and have the potential to overlap with state and local law enforcement organizations.

It is out of this delicate environment that fusion centers developed. Fusion centers are defined as a collaborative effort of two or more agencies that provide resources, expertise, and/or information to the center with a goal of maximizing the ability to detect, prevent, apprehend, and respond to criminal and terrorist activity (DHS, 2014). Predictably, with so many converging interests to serve, fusion centers have come under regular critical review. Privacy organizations have criticized the role of domestic intelligence centers, government accountability watchdogs and congressional reviews have targeted the viability and return-on-investment from fusion centers, and many practitioners of law enforcement and intelligence within the homeland security arena have questioned the efficacy and efficiency of fusion centers as information sharing hubs (Masse et al., 2007). Salvatore (2018) conducted three case studies on why fusion centers are not sharing intelligence and found 3 reasons namely: the absence of a standardized fusion center model, an insufficient concentration on counterterrorism as a mission, and underdeveloped or missing external agency partnerships The centers have their defenders, as well, and while much of what is written on fusion centers in professional and peer-reviewed journals could be interpreted as critical of at least one function they perform, there is also a consensus that the requirement they were created to fill is a crucial one (Coffey, 2015).

**Figure 1.**
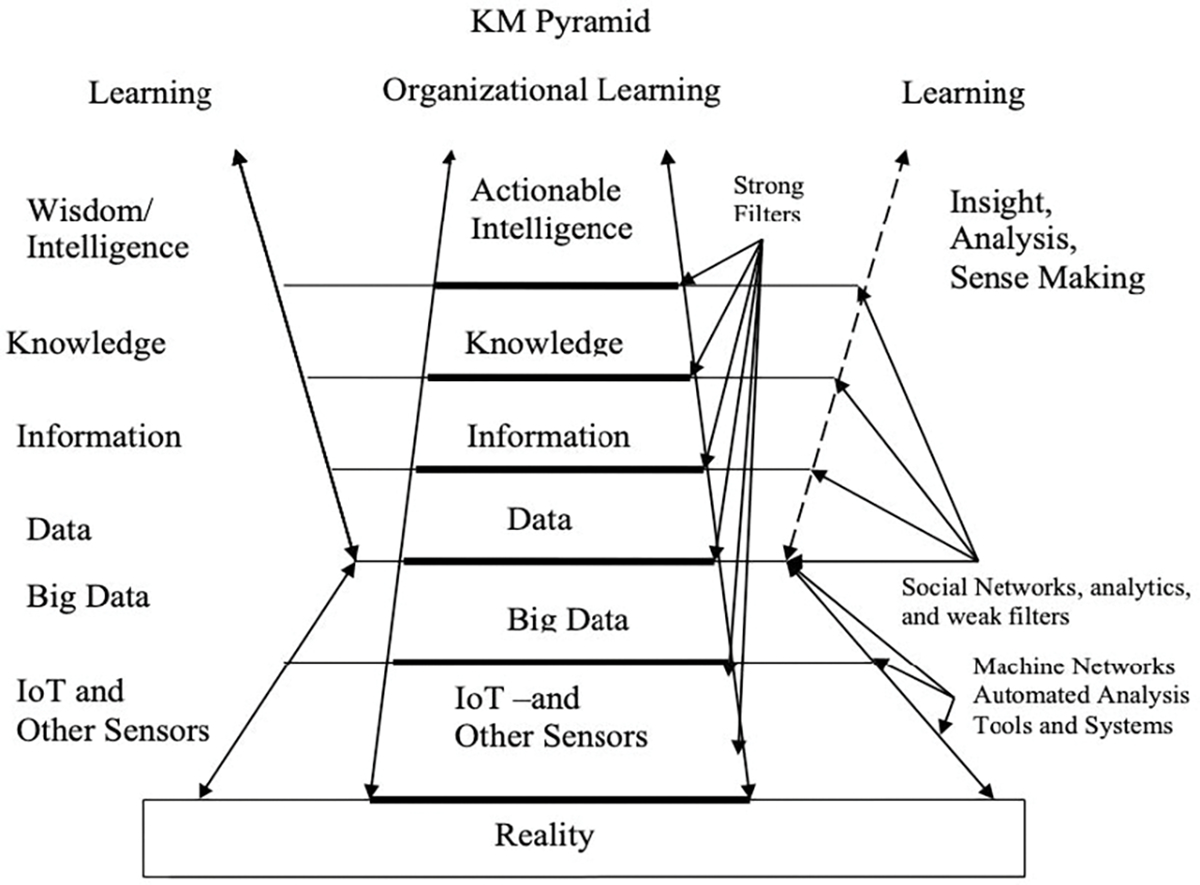
Jennex Revised Knowledge Pyramid (Jennex, 2017)

This paper addresses the research questions of what are the historical barriers to information sharing, and how can fusion centers become more efficient and effective in this role? These questions are researched using case study research on a Structured Analytic Technique exercise to observe information sharing barriers and proposes that applying the concepts of the modified knowledge pyramid (figure 1) (Jennex, 2017) would help fusion centers create a strategy to produce actionable intelligence and thus improve the effectiveness of the fusion center.

Figure 1 shows the hierarchy of knowing with the top level being actionable intelligence. It is the function of fusion centers to produce actionable intelligence. The hierarchy shows that to produce actionable intelligence, a fusion center needs to create filters that separates useful data, information, and knowledge from the vast amount of irrelevant data, information, and knowledge that has been collected. Figure 1 shows several processes that can be used to create these filters and the work of others, such as Dong and Srivastava (2013) focuses on techniques, in this case big data integration for applying these processes to creating these filters. See Jennex (2018) for more discussion on the model. This paper uses this model to guide formulation of a data fusion center strategy (discussed later in the paper).

## 2. BACKGROUND

As previously stated, state and local fusion centers were created from the feedback and insight of the 9/11 Commission Report, and the demonstrable need for formalized information sharing between federal, state and local entities. This proved difficult to accomplish in execution. Congressional studies, Department of Homeland Security annual reports, and academic work from security and intelligence professionals were all very blunt in identifying their weaknesses. Multiple attempts were made to establish fusion center guidelines and delineate a set of baseline capabilities, while acknowledging that fusion centers at the state and local levels were prone to develop in ways that seemed to fit a regional niche (DHS, 2016). A recurring theme in critiques is that while fusion centers were quick to adapt in ways that would add value, primarily at the local and regional level, the activities they undertook were not what would traditionally be defined as the “true fusion” of all sources of intelligence (Masse et al., 2007).

**Figure 2.**
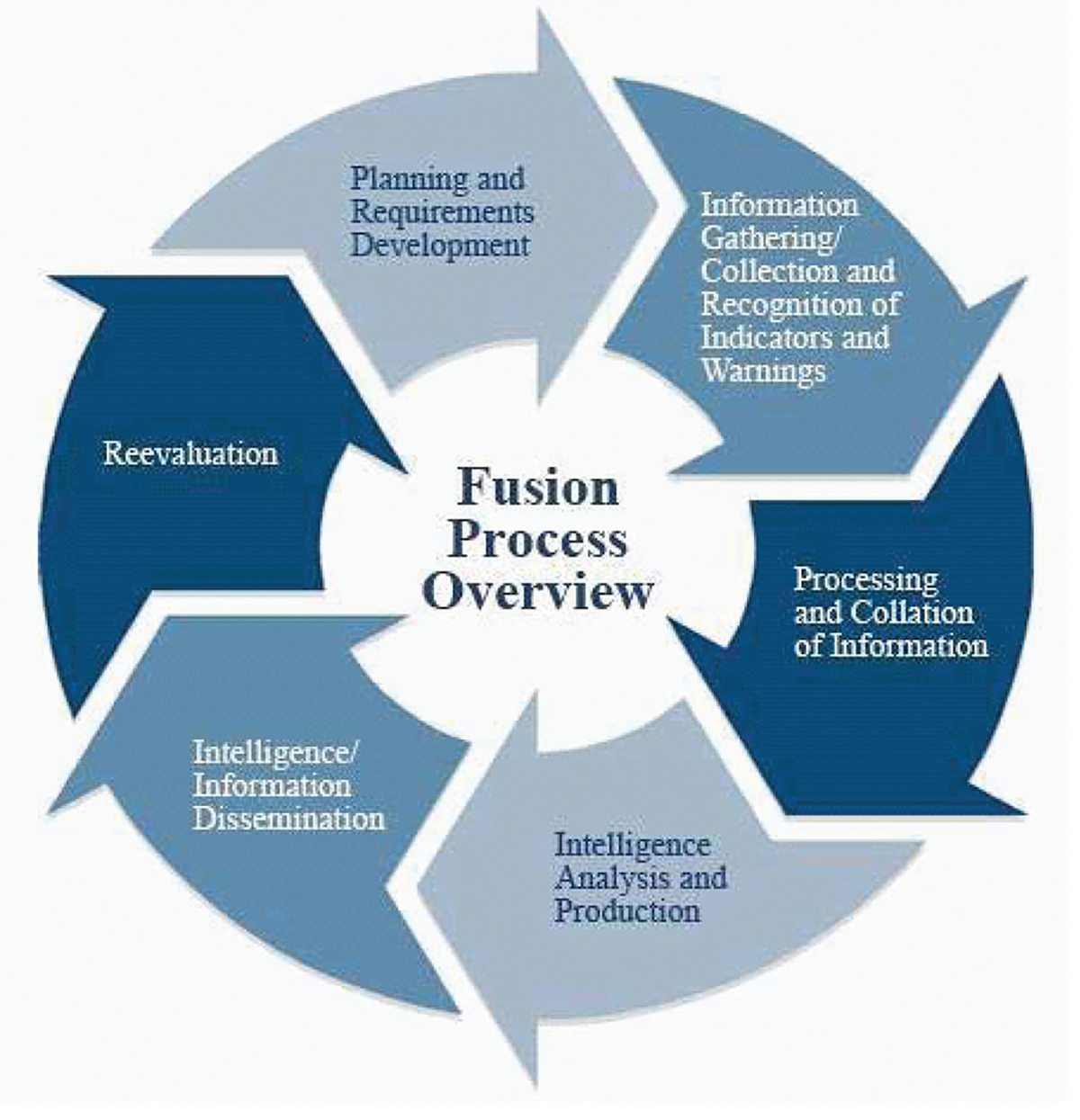
The Fusion Process Overview (ODNI, 2020)

The Department of Defense definition of fusion is a multilevel, multifaceted process dealing with the automatic detection, association, correlation, estimation, and combination of data and information from single or multiple sources (DHS, 2014). The Department of Justice, in establishing the guidelines for state and local fusion centers, characterized the fusion process by its ability to combine possibly uncertain, incomplete, and contradictory data, perhaps resulting in data or information of improved quality (DHS, 2014). Together, these concepts form the key function which state and local centers were created to perform, with the process illustrated by the diagram below (Figure 2), which amplifies the intelligence cycle.

Figure 2 demonstrates the perpetual activities undertaken by fusion centers, and places them into their proper order. Each step of this process can be done by organizations which contribute to fusion center efforts at their own facilities. The strength which fusion centers bring is the ability to conduct all these steps within the same physical location, decreasing the time the cycle takes to complete and thereby increasing the capacity to execute multiple cycles.

The most effective fusion centers are collocated with agencies or task forces fulfilling other existing functions, such as High-Intensity Drug Trafficking Areas or Joint Terrorism Task Forces. Layering fusion centers with existing multi-agency organizations was meant to leverage existing relationships to further improve information sharing. This led to fusion centers expanding from the initial roles of counterterrorism analysis into a more all-crime/all-hazards approach. Fusion centers added mission sets to fill observed gaps within their regions, supporting investigations, de-conflicting local law enforcement operations, or supporting special events, all of which have exponentially increased the amount of information that they consume. One thing all these functions require, however, is effective use of this information. This is where the concepts of knowledge and information management come into play, which facilitate the following analysis to produce intelligence. With as much data and raw information as is available, a method to triage, identify information of value, and disseminate that information is vital.

Fusion centers support the drive for evidence-based decision making. Figure 1, the revised Knowledge Pyramid, shows that technologies gather and analyze observations of reality faster than and in amounts greater than what humans can process as the filters that support the creation of actionable intelligence (Jennex, 2017). This model provides value by showing relationships between technologies and decision support artifacts (Internet of things, big data, data, information, knowledge, intelligence) and by providing a model that assists in creating a strategy for selecting and managing technologies to create actionable intelligence for immediate decision making.

The model assists fusion center managers in planning and creating a knowledge management (KM) strategy to guide fusion center activities. Advances in Knowledge Management (AKM) strategy is a statement of how knowledge will be captured and used. Fusion centers need to have a KM strategy for managing the layers of knowing and technologies in the revised knowledge pyramid. Jennex (2010; 2012) found that having a KM strategy improved performance through better decision making; and identified the components needed in this strategy. The basic components of a KM strategy can be generalized and made useful for managing knowledge pyramid activities including:

- Identification of users of the knowledge pyramid layers and transformation processes.
- Identification of actionable intelligence needed to support decision making.
- Identification of sources of big data, data, information, and knowledge.
- Identification of big data, data, information, and knowledge to be captured.
- Identification of how captured big data, data, information, and knowledge is to be stored and represented.
- Identification of technologies to be used to support capturing and processing big data, data, information, and knowledge.
- Generation of top management support.
- Establishment of metrics for big data, data, information, and knowledge use.
- Establishment of feedback and adjustment process on the effectiveness of actionable intelligence use.

The goal is to add a top down strategy approach based on the decisions to be made and identifying the technologies and decision support components needed, to the bottom up data discovery process currently used. Additionally, the more focused the strategy the stronger the filters that are created blocking big data, data, information, and knowledge not needed to support decision making.

## 3. METHODOLOGY

In preparing this paper, the authors utilized official reviews and Congressional Research Study reports discussing the challenges facing fusion centers as they have matured in the years since the 9/11 terrorist attacks. The key arguments detailing inefficiencies and perceived weaknesses were then viewed and analyzed from a KM lens, with the resulting observations leading to a recommendation for improving the effectiveness on how the massive amounts of data are processed, disseminated, and used. This paper also uses a Structured Analytic Technique exercise as described by Heuer and Pherson (2015) as a case of a method that fusion centers could regularly implement to specifically address the challenges of knowledge sharing.

**Figure 3.**
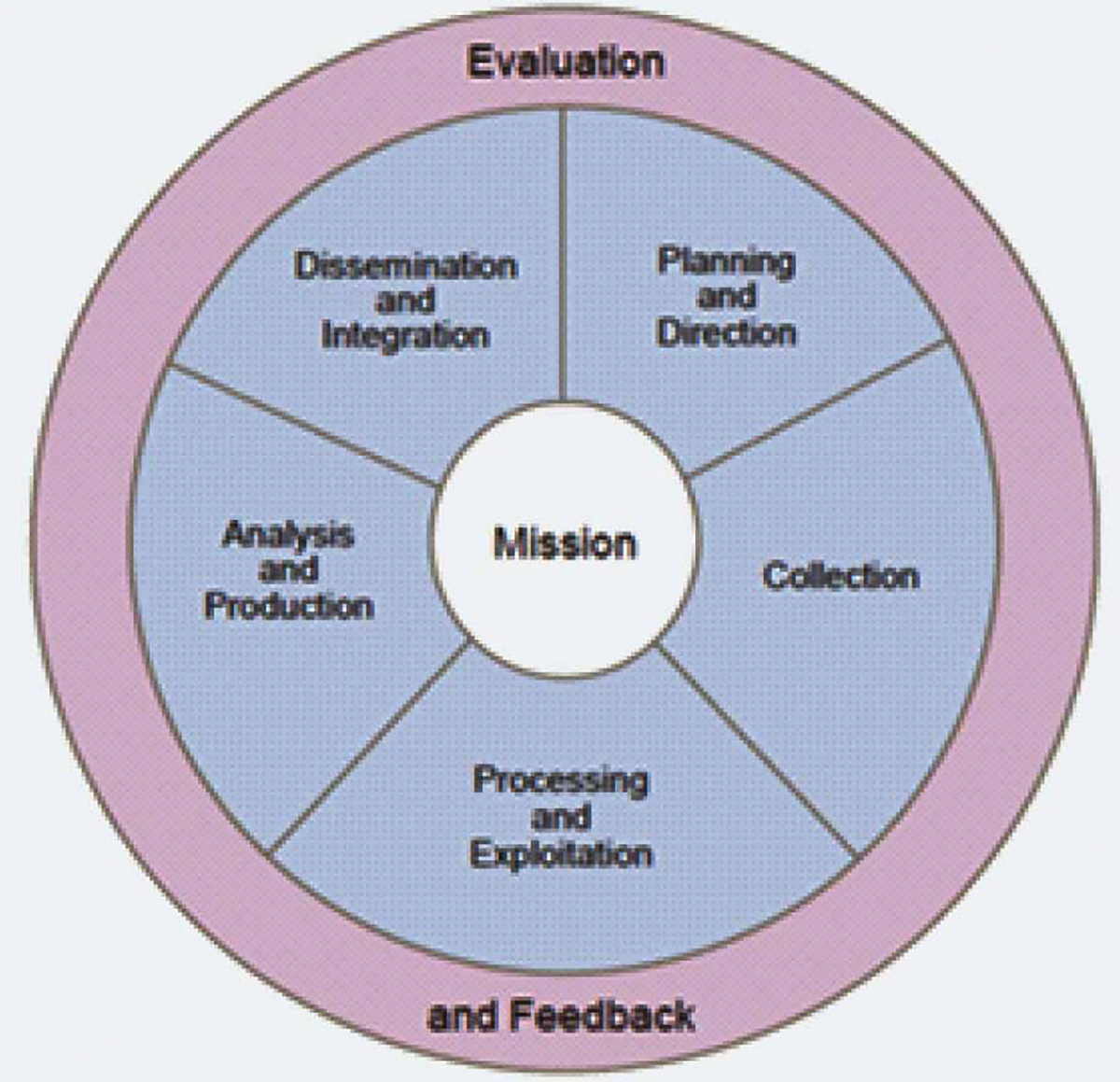
The Intelligence Cycle (DoD, 2013)

The Structured Analytic Technique (SAT) is presented to demonstrate and discuss how the analysts approached the data in this setting and utilized a flexible set of structured analytic options to transform that data into information, capture it as knowledge, and press it forward as actionable intelligence in line with the KM pyramid in Figure 1. The participants in this case were members of regional security committee within the San Diego public sector representing federal, state, and local law enforcement agencies. The SAT exercise was held in San Diego in the summer of 2018. The SAT analytic scenario was to assess the likelihood of three distinctive threat vectors occurring within the region. Reporting that originated from both local and federal law enforcement organizations was reviewed, analyzed, and interpreted by the participating analysts. During this single day event, the analysis led the participants to identify gaps, create intelligence and information requirements, and conduct analyst-to-analyst coordination among adjacent organizations with sympathetic interests in the security of their region. These efforts simultaneously completed several steps of the intelligence cycle, depicted in the diagram shown in Figure 3.

The approximately 20 participating members represented six organizations from the local government level to federal law enforcement agencies. Eight of the participants were also representatives from their organizations to the security committee, while the remaining analysts were brought in from the member organizations to provide additional experience and different perspectives.

Participants were split into three similarly sized teams, each focused on one aspect of the scenario. Analysis material comprised of threat reporting and crime data provided by the participating member organizations from the prior two years.

The first team conducted structured brainstorming to generate a range of hypotheses (Heuer and Pherson, 2015) and quickly surmised that there were significant gaps in the available reporting that hindered the ability to create relevant and specific hypotheses based on the evidence. However, the divergent thinking process led to the identification of a series of conditions that existed which lacked any of the type of reporting seen. The technique of creating a list of descriptive indicators (Heuer and Pherson, 2015) was then used to create a specific and descriptive list of expected indicators for a number of distinct scenarios which were translated into and captured as intelligence requirements. By identifying the conditions that enabled the activities noted in the relevant reporting, the team was able to extrapolate additional scenarios and create a series of observable indicators to prove or disprove hypotheses.

The second team conducted a thorough analysis of competing hypotheses against their problem set, a useful tool to combat the phenomenon of satisficing, or going with the first answer that seems to be supported by the evidence (Heuer and Pherson, 2015). Analysis of competing hypotheses requires analysts to identify and then try to refute as many reasonable hypotheses as possible using the full range of data, assumptions, and gaps that are pertinent to the problem at hand (Heuer and Pherson, 2015). By trying to disprove an analytical hypothesis, it forces the participants to go against their own assumptions and look beyond any potential cognitive biases by looking specifically for reporting or indicators which support a single, mutually exclusive theory while discounting those which support multiple, mutually-exclusive theories.

The third team used a quadrant hypothesis generation model to prioritize responses to scenarios stemming from two primary drivers. This tool is used when the situation is affected by relatively few driving forces, and identifies four potential scenarios that represent the extreme conditions for each of the two major drivers (Heuer and Pherson, 2015). This enabled them to extrapolate the unique indicators which would point to one of the four potentialities.

## 4. FINDINGS

While the initial goal of the session was not fully met (an assessment of the likelihood of several threat vectors occurring in the region), there were a number of valuable takeaways and results. On the tactical level, multiple gaps in the collective knowledge regarding the handling of security violations at critical infrastructure sites were specifically identified and captured, along with the creation of observable indicators that would answer those gaps. At the organizational level, analysts from the participating agencies had the opportunity to expand their perspective on issues they work on as a daily part of their jobs, as well as creating ‘boundary-spanning’ relationships with their peers in adjacent agencies. Individually, each analyst expanded their knowledge of both the capabilities and limitations of each participating agency, improved their understanding of their own problem set, and brought back to their agency both the procedural knowledge to recreate this process on additional topics and the specific knowledge of the problem we analyzed as a group.

The execution of the SAT effort accomplished more than just a transfer of knowledge across participating agencies. The benefits were threefold: data was presented that had not previously been viewed by all parties; capabilities, strengths, and weaknesses of participating agencies were shared and discussed amongst the group; and relationships were fostered to further the sharing of both information and knowledge on an analyst-to-analyst level into the future. A previous key critique (Rollins, 2008) was that little ‘true fusion,’ or analysis of disparate data sources, identification of intelligence gaps, and pro-active collection of intelligence against those gaps which could contribute to prevention is occurring. The analytic exchange which occurred in the course of a single day accomplished all of these tasks on a regional level. New data was shared among all members, formal analytical processes were executed to fuse the data into information, and then the information into intelligence, and the resulting intelligence was further examined to identify gaps in knowledge and craft requirements to drive future collections and operations.

Leveraging boundary-spanning analysts with overlapping interests, the intelligence produced by the group was of far greater value than the sum of its parts. Those participating analysts returned to their agencies at the end of the day with actionable information that could be injected into the specific collection management operations of their own organizations. Additionally, they could then brief their leadership on the complementary and supporting activities of their partner agencies to produce a greater situational awareness of friendly efforts along similar lines of effort.

The analysis was recorded in an after-action report (AAR) that captured the step-by-step process each team took to approach the problem they faced. The AAR recorded the specific inputs and ideas generated by each group, to include hypotheses and theories, allowing supervisors or analysts looking at the same issues to retrace the steps of the exercise. The intelligence portion of the effort was captured in a brief for the regional security committee’s executive steering group. The brief highlighted to the committee’s leadership key concerns and information gaps identified by the analysis, as well as recommended strategies for educational outreach to improve the amount of relevant threat reporting coming in. At the brief, the lead analysts were able to present additional efforts undertaken by participating agencies which were spurred by the analytical effort, demonstrating the value of ‘actionable analysis.’ In one case, a specific gap identified by the collaborating analysts initiated an operation within a participating law enforcement organization that resulted in the arrests of individuals who had been attempting to exploit the very vulnerabilities the group highlighted.

With the resulting intelligence taken for action by participating agencies immediately after its generation, there was no time lost between the conduct of the analysis and waiting for a formal product to be published. The formal, structured process of this method captures every input, as well as the thought process and interpretation of data used to form the analytic line, easily allowing supervisors or objective observers to audit and review the results. This ease of auditing acts as quick balance to the lengthy review process conducted for written analytical products. Focusing on the immediate applicability of the ingested information speeds up the intelligence cycle functions by combining processing and exploitation with analysis and production, so that the resulting dissemination and integration can directly be utilized to spur action (See figure 3). Those actions then produce new inputs that drive evaluation and feedback to let the analysts know whether their work has been useful and help to answer gaps that were previously identified.

This process is the same that occurs in any organization doing work in the intelligence field, however, it was focused and streamlined within the fertile microcosm of a collaborative and structured analytical setting. Absent only the Intelligence Cycle function of collection, the SAT session facilitates the timely translation of data first into information and then directly into intelligence (Bundy, 1959). These concepts will further be explained in the next segment, as the successful and replicable processes demonstrated by executing collaborative SATs are further unpacked in the light of KM.

## 5. DISCUSSION

Fusion Centers conduct or contribute to a statewide and/or regional risk assessment that identifies and prioritizes threats, vulnerabilities, and consequences at regular intervals (Global Justice Information Sharing Initiative, 2008). These regular assessments take several forms, and also offer ideal opportunities for cross-organization coordination and collaboration. This paper presented a case where a SAT process was used by a multi-agency organization to improve knowledge sharing and increase effectiveness of actionable intelligence generation. It is anticipated that other fusion centers can leverage their combined analytic strength through focused SATs to craft timely, sound, and relevant intelligence. The result would be ideal for both independent agency operations and shared situational awareness. SAT addresses several of the core knowledge sharing and intelligence generation issues in KM to improve organizational effectiveness and decision making (Jennex, 2005). Among these are the knowledge sharing barriers of lack of trust and fear of loss of control over generated knowledge (Cleveland and Ellis, 2015). SAT addressed these issues by getting teams together in a manner that generated team trust and got them to produce recommendations that allowed all team members to claim ownership.

Fusion center effectiveness is measured using metrics such as production numbers and product types (DHS, 2018). Many studies at the Congressional level have identified that most of the reporting being completed is redundant or overcome by events by the time it has been released (Rollins, 2008). Additionally, because the funding is decided locally while the rating occurs federally, there is a disconnect between what fusion centers do on a daily basis and what they are graded for (ODNI 2020). KM provides an excellent framework and lens through which to view the functions of fusion centers (Records, 2005), and a more practical model for realizing the true value and return on investment which fusion centers can offer. By exploring key concepts such as the sharing of information, transforming it into knowledge, and capturing it as actionable intelligence at the organizational level, the benefit of efforts such as SAT become clear. When SAT is viewed in light of Figure 1, the value of each step is apparent and demonstrates progress toward improved organizational effectiveness.

The SAT case in this paper is presented as a mechanism for improved sharing of actionable intelligence at the local and regional level. Previous discussion above has laid out the critiques against fusion centers, offering reasons why the deceptively simple concept of information sharing is so difficult to execute. Fortunately, a great deal of research has been conducted into the academic concepts which make this process work and work effectively. KM researchers have identified three distinctive perspectives for individuals sharing information for the public good: they share knowledge without the need for reciprocity (cooperators), they feel obligated to share their knowledge (reciprocators), or they take knowledge for granted (free riders) (Snyder et al., 1999; Kim et al., 2006). The fusion center plays the role of the cooperator in sharing as much information as possible and facilitating the collaboration amongst all participants, who are reciprocators. Additionally, KM explains why SAT improves knowledge sharing by looking at the concepts of affective organizational commitment reciprocity, and enhanced reputation. Affective organizational commitment is improved when members get a feeling of belongingness with the organization. Fusion centers are multi-organizational resulting in lower feelings of belongingness. SAT increases belongingness by having the fusion center members work together in multi-organizational teams (Dey and Mukhopadhyay, 2018). Reciprocity increases knowledge sharing as members are more likely to share when they feel other members will also share. Reciprocity is improved when the employees build relationships, SAT, does this through its team approach (Todorova and Mills, 2018). Enhanced reputation increases knowledge sharing as the sharer perceives it as a reward for sharing. SAT, through its team approach, allows its members to generate reputation through their participation in sharing (Todorova and Mills, 2018). Finally, organizational effectiveness has been shown to be improved when knowledge sharing is improved (Crhová and Matošková, 2018) so a SAT improves fusion center effectiveness by improving knowledge sharing.

Additionally, KM views the analytical collaboration created by fusion centers is as de facto communities of practice (CoP). CoPs are groups of people informally bound together by a shared practice and passion for a joint enterprise (Koeglreiter et al., 2018). CoPs are a social construct, characterized as a social group that shares common objectives but which is not necessarily structured as an organizational unit (Koeglreiter et al., 2018). Both definitions fit the purpose of the type of collaboration that fusion centers were created to facilitate, as well as the cooperation to which SATs lend themselves.

When viewed as regional CoPs, fusion centers are ideally placed to fill the critical role of an organization champion within the collective group, where organizational champions (cooperators) bring sufficient knowledge and foster a rich intellectual environment are one of the key success factors of CoPs (Cleveland and Ellis, 2015). The challenge in enabling the fusion center to fill the role it was designed for is establishing the correct context to gather and form a CoP, as at any given time a fusion center could realistically be dedicating resources and analysts toward counterterrorism issues, criminal issues, and all-hazards issues simultaneously. Each focus could generate its own CoP, with that CoP providing a collective knowledge-base that varying members can access freely and to which they can contribute with or without expecting benefits (Cleveland and Ellis, 2015).

To keep the CoP viable and engaged requires more than just bringing like-minded and experienced law enforcement organizations and analysts together. KM researchers have revealed correlations that the fraction of cooperators is positively related to the total knowledge contribution and to the reciprocity level, while the reciprocity level positively affects knowledge contribution (Cleveland and Ellis, 2015). This reflects the ability of cooperators to influence the overall sharing of information and increase the total amount of information shared by participants in a CoP. If participants do not see value in sharing or are not influenced to contribute some effort to the group, the overall effectiveness of a CoP goes down in generating collective knowledge or intelligence. This is the beauty of collaborative SAT efforts: a single cooperator can generate the raw data and information which participating reciprocators and even free riders can analyze in a cooperative setting to generate new knowledge to the benefit of all.

A single successful session can generate demonstrable results, and the credit can be shared among all participants. The additional benefit to holding such sessions is that knowledge sharing research suggests individuals are more likely to share expertise inter-organizationally via face-to-face, than through other indirect or impersonal methods (Cleveland and Ellis, 2015). Direct and interpersonal settings serve to break down many of the organizational and systemic barriers to information sharing and can facilitate greater information sharing via the systems that are required to conduct any mission worked by fusion centers.

The meticulously structured nature of SATs allows future repetitions of such efforts that look at evolving threats and build upon the efforts of the analysts who have conducted these processes before them. Identifying any initially held cognitive biases, and the circumstances which led to any assumptions made by previous analysts gives future iterations a foundation upon which to build their efforts. It also creates a body of notes for posterity that can provide future analysts with thought-provoking hypotheses or theories that had been discounted but could prove viable in light of new and emerging information. In this sense, the process blends even more into the definition of a knowledge management success, “reusing knowledge to improve organizational effectiveness by providing the appropriate knowledge to those that need it when it is needed (Department of Defense, 2013).

The Revised Knowledge-Knowledge Management Pyramid (figure 1) depicts how raw inputs are examined in light of previous experience and learning, revised with research and analysis, and combined to become knowledge and organizational learning. This provides the ideal context for describing the value of collaborative SAT efforts, while the former depicts quite simply the goals of the intelligence cycle.

In the context of a SAT effort, the social network on the periphery of the figure 1 is indicative of the participating analysts, and their unique perspectives and experiences. As the observations of reality are interpreted by the sensors (collection) which report the raw data, the application of the intelligence cycle transforms the inputs from data to information, and eventually to actionable intelligence. As the process occurs, in addition to the arrows approaching organizational learning in the center, individual learning is depicted by the arrows radiating outward. Thus, as the knowledge is created that benefits the group, so too is knowledge that benefits each participating analyst, growing both the whole and the sum of its parts simultaneously.

Figure 1 includes additional levels of filters and sensors representing the recognition that technology has enabled the collection, aggregation, and filtering of data at a staggering level. The Internet of Things (IoT), the ability to ingest unstructured data from multiple unrelated databases, and machine learning applications have driven the concept of big data with the goals of identifying intelligence for evidence based decision making, transforming intuitive based decision making to evidence based decision making, and pushing decision making to lower levels of the organization (Jennex, 2020). Figure 1 demonstrates the potential that could be reached by a more unified and organized approach to engaging in SAT efforts, and hints at the results that could be achieved. As the event was conducted using limited inputs from the participating organizations, it is a testimony to the process that successful results were returned.

With the resulting efforts captured both in intelligence products and AARs, analysts can review the outputs as well as how the inputs were treated to reach their analytical conclusions. As organizational learning is the consequence of this process, knowledge and wisdom are captured at the analytical hub of the activity. With the fusion centers serving as the apex of this process, the wisdom generated through communal analysis is available for all participating agencies.

The value generated by the structured sharing of information in this manner plays to the strengths which numerous studies identified in fusion centers, to include the formal and informal boundary-spanning connections at the analyst level. It counters many of the weaknesses including lengthy deliberate production timelines and barriers to information sharing. The additional benefit of the process is that it is simple and repeatable, with no specialized knowledge required above basic analyst training. These concepts are introduced at the novice level, but far too few analysts ever execute them in their positions. By increasing the use of these SAT efforts on a national level across the National Network of Fusion Centers, many more regional issues could be engaged and more available data could be transformed into actionable analysis.

Additionally, fusion centers have expanded their focus capacity. While this was initially met with friction, and was cast in a negative light by official Congressional and Department of Homeland Security reviews, this shift demonstrated an organic settling of fusion centers into a needed role to provide a regional context for both criminal and all-hazards threat information. The Department of Homeland Security Inspector General Report (DHS, 2008) that arrived at the same conclusion, that up until that point, some intelligence products could better meet state and local needs. These observations drove a natural migration where fusion centers shifted their focus to better fill local and regional requirements, particularly as state and local governments began to pay an increasing portion of fusion center operating costs.

Between these efforts and others, fusion centers began to mature around 2010, demonstrating many successes both in contributing to the disruption of terrorist plots at the local and regional level, as well as providing valuable all-crimes, all-hazards threat support to state and local law enforcement jurisdictions. However, many challenges to reaching the initial information sharing goals remain. Chief among them are the difficulty of conducting true intelligence fusion processes and disseminating jointly authored analytical products in a timely manner. As critique after critique identified, too often fusion centers failed to produce analytical products that kept up with the speed of relevance (DHS, 2010).

**Figure 4.**
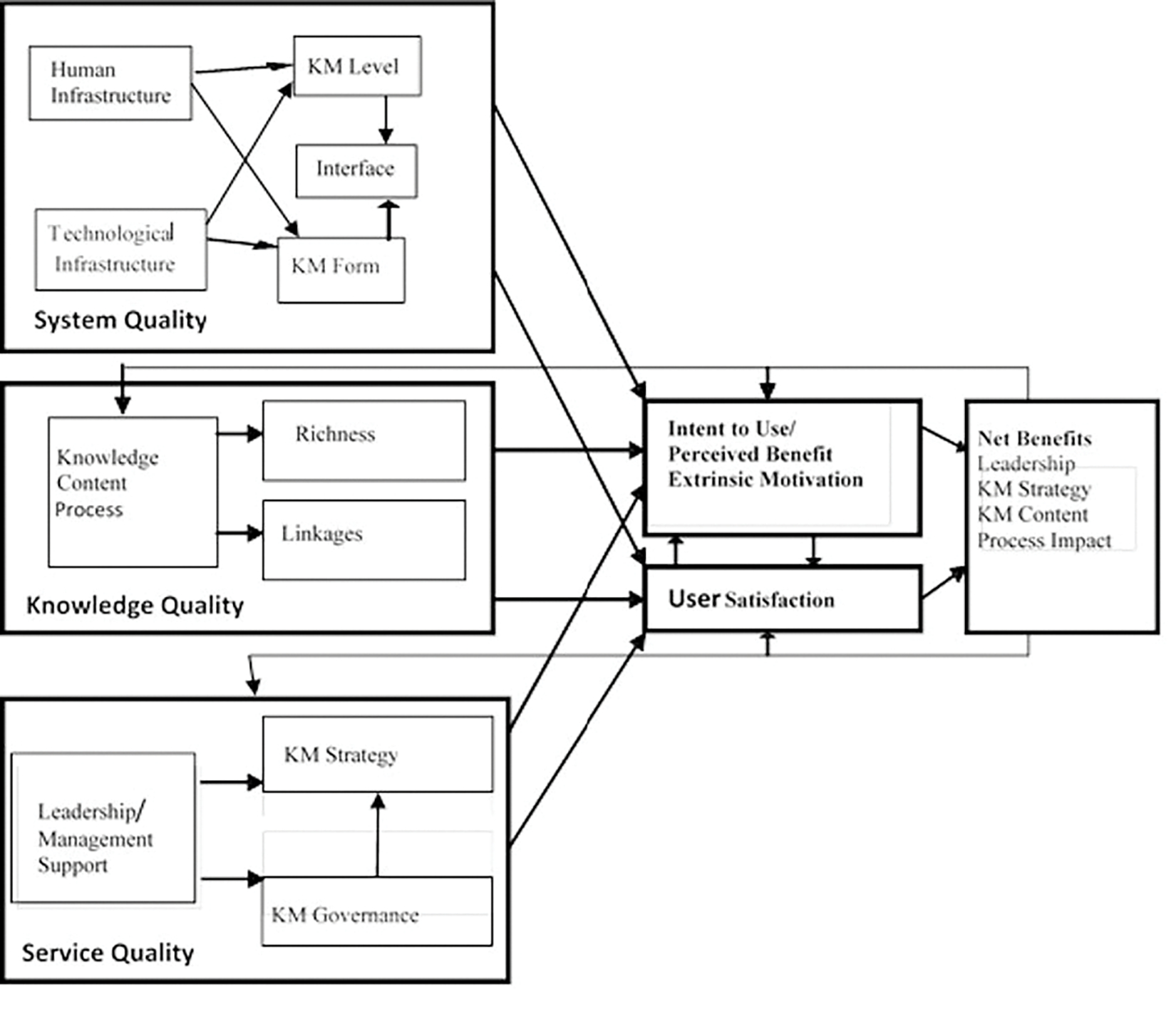
Re-specified Jennex Olfman KM Success Model (Jennex, 2020)

One aspect of that issue missing from this paper is the study of the information systems (IS) utilized by these agencies to facilitate knowledge sharing and collaborative analysis to produce intelligence. The primary existing networks have been identified as the Homeland Secure Data Network for classified information sharing, and the Homeland Security Information Network for controlled but unclassified information sharing between federal, state, and local partners. Discussion of these IS and their contributions to or detractions from the issue of information sharing and collaborative analysis have been avoided deliberately to focus on the human factors which fusion centers can largely control or influence. However, these systems do have an impact on KM and knowledge sharing as is shown by Figure 4 where the impact of technical resources influences the success of KM.

The re-specified Jennex-Olfman KM Success Model, Figure 4, identifies three primary driving factors for successful KM efforts. Those drivers are system quality, or the technological resources and human infrastructure devoted to the effort; knowledge quality, the usefulness and accuracy of the content and its ability to help users in performing their duties; and service quality, the support of the effort demonstrated by leadership/management support (Jennex, 2020).

This model offers fusion centers insight that supports that improving fusion center effectiveness also needs IS sufficient to support effective knowledge sharing and applies directly to conducting successful SAT events. Another takeaway from Figure 4 is the equal importance of leadership buy-in to the process, demonstrated by having direction from the top of the organization that appropriates the resources needed for KM as well as creates and maintains the knowledge sharing, knowledge using culture necessary for KM to succeed (Jennex, 2020). Placing this concept on equal footing with both the quality of the processed knowledge and quality of the systems and analysts conducting the efforts reflects the importance that organizational culture has on achieving successful information sharing results.

Finally, further research analyzing the KM Success Model through partial least squares – structural equation modeling (PLS-SEM) (Kundapur and Rodrigues 2017; Srivastava and Joshi 2018) has found that while Figure 4 shows the precursors to KM success, they also form measures of KM success. With leadership support, knowledge quality can likewise be improved to feed the process. By increasing the amount and quality of data available to be analyzed through incorporating the myriad open-source sensors and resources available to fusion center partners, and utilizing technological filters including artificial intelligence algorithms to parse the data into digestible packets, SAT efforts could receive a significant head-start in the process of producing actionable intelligence. Finally, leadership support can ensure technology resources are available for a trained and eager cadre of analysts to fully utilize in their collaborative efforts and set the mission up for success, where recognition of KM success is getting the right knowledge to the right people at the right time (Jennex et al., 2016). A further suggestion is that this research can be used to create additional metrics for measuring fusion center success focused on leadership, content, and center focus to be used in addition to the previously discussed process measure.

## 6. CONCLUSION

The key conclusions of this paper are that knowledge sharing and fusion center effectiveness can be improved by using SAT. SAT increases affective organizational commitment, reciprocity, and enhanced reputation by fusing the members of the fusion center into teams and creating a CoP. Fusion center effectiveness is increased as it has been shown that improving knowledge sharing will improve organizational effectiveness. Additionally, SAT performance can be improved by improving the IS used to better support the human factors of affective organizational commitment reciprocity and reputation enhancement.

The net benefits of a SAT effort producing actionable intelligence are clear to the decision maker, and can directly affect the environment in a timely manner. Timely results are at a premium in resource-constrained environments. When funding for national security efforts is viewed as a zero-sum game, each agency with a role in this mission must seek both efficiencies and successes to maintain or improve funding levels which come at the expense of another organization in the field. This is one of the additional drivers of joint intelligence products, and why contributing organizations benefit from successful collaborations. When bureaucratic oversight processes delay the results to the point of irrelevance, additional practices are required to generate timely and useful intelligence that also benefit multiple agencies. Thus, organized and focused SAT efforts, conducted under the National Network of Fusion Centers, are that alternatives.

SAT is not a silver bullet to overcome every hurdle to knowledge sharing, and it does require an amount of experience and initiative to properly execute. The drivers of the previously explored KM success model should be enacted while building that experience. If this model were to become institutionalized at the fusion center level, across the National Network of Fusion Centers, both the capability and capacity to conduct truly effective SAT efforts would quickly expand. This process is likewise not a response to every critique levied at fusion centers for failures in information sharing, but it is a proposed solution to strengthening local and regional relationships between analysts, bringing like-minded professionals together to look at shared problems, and creating the types of opportunities where boundary-spanning activities can occur. In short, it is that effort to use many of the things that are best in fusion centers to counter some of those issues which outsiders view as wrong with fusion centers.
